# Surgical treatment of pressure injuries in children: A multicentre experience

**DOI:** 10.1111/wrr.12964

**Published:** 2021-09-02

**Authors:** Marco Pignatti, Salvatore D'Arpa, Nathalie Roche, Federico A. Giorgini, Irene Laura Lusetti, Concepcion Lorca‐Garcia, Giorgio De Santis, Beatriz Berenguer

**Affiliations:** ^1^ Plastic Surgery IRCCS Azienda Ospedaliero‐Universitaria Sant'Orsola di Bologna Bologna; ^2^ DIMES University of Bologna Palermo; ^3^ Plastic and Reconstructive Surgery La Maddalena Cancer Center Palermo Italy; ^4^ Department of Plastic and Reconstructive Surgery Ghent University Hospital Ghent Belgium; ^5^ Plastic Surgery University of Modena e Reggio, Policlinico di Modena Modena Italy; ^6^ Pediatric Plastic Surgery Hospital General Universitario Gregorio Marañón Madrid Spain

**Keywords:** children, perforator flap, pressure injury, pressure sore, recurrence

## Abstract

Pressure injuries (PI) are infrequent in paediatric patients, prevalence estimates ranging from 1.4% to 8.2%, and reaching values as high as 43.1% in critical care areas. They can be associated with congenital neurological or metabolic disorders that cause reduced mobility or require the need for medical devices. In children, most pressure injuries heal spontaneously. However, a small percentage of ulcers that is refractory to conservative management or is too severe at presentation (Stage 3 or 4) will be candidates for surgery. We retrospectively reviewed the clinical history of paediatric patients affected by pressure injuries from four European Plastic Surgery Centres. Information was collected from clinical and radiology records, and laboratory reports. An accurate search of the literature revealed only two articles reporting on the surgical treatment of pressure injuries in children. After debridement, we performed surgical coverage of the pressure injuries. We report here our experience with 18 children aged 1–17 years, affected by pressure injury Stages 3 and 4. They were successfully treated with pedicled (17 patients) or free flaps (1 patient). The injuries involved the sacrum (6/18 patients), lower limb (3/18 patients), thoracic spine (2/18 patients), ischium (3/18 patients, bilateral in one patient), temporal area (3/18 patients), hypogastrium (1/18 patients) and were associated to medical devices in three cases. Flaps were followed for a minimum of 19 months and up to 13 years. Only two patients developed true recurrences that were treated again surgically. Pressure injuries are infrequent in children and rarely need surgical treatment. Pedicled flaps have a high success rate. Recurrences, contrary to what is reported in the literature, were rare.

**Abbreviations**
ALT: anterolateral thigh
EPUAP: European Pressure Ulcer Advisory Panel
IGAP: inferior gluteal artery perforator flap
NICU: Neonatal Intensive Care Unit
NPIAP: National Pressure Injury Advisory Panel
NPUAP: National Pressure Ulcer Advisory Panel
PPPIA: Pan Pacific Pressure Injury Alliance
PI: pressure injury
SGAP: superior gluteal artery perforator
TPF: temporo‐parietal fascia flap

## 1 INTRODUCTION

A pressure injury (PI) is defined as localized damage to the skin and underlying soft tissue, usually over a bony prominence or related to medical or other devices.^1^

In 2016, the National Pressure Ulcer Advisory Panel (NPUAP).^1^ suggested that the term injury be used instead of ulcer and that the stages be denoted using Arabic rather than Roman numerals. The NPUAP's staging system that describes the extent of tissue loss and the physical appearance of the injury caused by pressure and/or shear, progressing from Stages 1–4, has been widely adopted internationally and has become the basis for treatment and comparison of outcomes. A particular type of PI's are the unstageable pressure injuries, full‐thickness lesions in which the base is obscured by slough and/or eschar and whose correct identification can be challenging.^1^

Detailed artwork describing the appearance of different stages, as agreed upon during the Consensus, can be found in reference.^1^

PIs can be painful, can be complicated by infection, impact negatively on the quality of life, and heal with difficulty, despite the availability of many therapies and preventive measures, none of which has been demonstrated to be superior to the others.^2^

Although they are more frequent in the adult, and especially in the geriatric population, pressure injuries can develop also in children.^3^

Aetiology is variable, but the most important role is played by medical conditions that cause reduced mobility or require the need for medical devices, that are responsible for PIs in 38.5%–90% of paediatric cases. Patients with this type of PI tend to be younger.^4^, ^5^

Visscher and Taylor^4^ evaluated Neonatal Intensive Care Unit (NICU) patients between 2007 and 2009. They found that nearly 80% of the PIs were associated with devices, and more than 90% of device‐related PIs occurred in the premature infants.

Premature birth, spina bifida, congenital neurological or metabolic disorders, heart disease, often associated with poor vascularization, decreased sensation, friction of skin against bone and shear of skin and bone sliding across one another, and malnutrition^6^ increase the risk of developing a PI. The Braden Q and Braden QD Scales are the most commonly used instrument to predict PI risk in paediatric patients,^7^ although a recent Cochrane review^8^ did not find enough reliable evidence from the published studies to suggest that the use of structured and systematic pressure ulcer risk assessment tools reduces the incidence, or severity of PI's.

The reported data on the incidence and prevalence of these lesions are not uniform.

Reliable numbers on the prevalence of PIs in children can be found, among others, in a large study including 39,984 patients 1 day to 18 years old treated in 678 paediatric acute care units of 271 US hospitals. The data, collected in 2012, had been submitted to the National Database for Nursing Quality Indicators and indicated the PI prevalence to be 1.4% with a prevalence of hospital‐acquired pressure ulcers of 1.1%. The highest rate was present among children aged 9–18 years (1.6%) and the lowest among patients 1 to 30 days of age (0.72%).

As expected, the highest prevalence was found in critical care units (3.7%) and paediatric rehabilitation units (4.6%), while hospital‐acquired PIs were more rarely observed in general paediatric wards (0.57%). Most of the hospital‐associated PIs were Stage 1 and Stage 2 (65.6%).^9^

A much higher prevalence of paediatric, PIs was found by a 1‐day cross‐sectional study performed in Switzerland, including 412 patients aged 0–18 years. The overall prevalence was 35% and most patients with PIs (80%) had Stage 1 ulcers.^10^ PIs can also affect neonates. Low gestational age, congenital disorders, in particular myelomeningocele, cardiac defects, genetic and metabolic syndromes, all represent risk factors for the development of PIs. The need for respirators or other devices significantly increases the risk. In a 2‐year‐long prospective study, PIs were device‐related in 80% of term new‐borns and in 90% of premature babies.

Compared with adults, paediatric patients require special consideration, protocols, guidelines, and standardized approaches to PI prevention. Nutritional support and pressure redistribution appear to be of uppermost importance.^11^ The guidelines, regularly published by the National Pressure Advisory Panel white paper, encourage the adoption of standardized efforts of interprofessional teams to successfully prevent and treat PIs in paediatric patients.^12^

In an intensive care unit, the hospital‐acquired PI rate decreased from 30% to 0% as a consequence of the nurses' dedication. In children, most of the pressure ulcers heal spontaneously or with medical help or minimal surgical intervention.^13^

However, a minority of ulcers that is refractory to non‐operative management or is too severe at presentation (NPUAP Stage 3 or 4) will be candidates for surgery.^14^

An accurate search of the literature revealed only two articles reporting on the surgical treatment of PIs in children.^14^, ^15^

We report here our experience with a retrospectively evaluated series of paediatric patients who underwent surgical treatment of PIs.

## 2 METHODS

We reviewed the records of 18 patients who underwent flap reconstruction for pressure injuries from 2007 to 2017 at four European Plastic Surgery Departments. The principles outlined in the Declaration of Helsinki have been followed. Clinical data of patients were collected retrospectively and included age, anatomical site, comorbidities, PI stage, medical and surgical treatment, recurrence and other complications.

### 2.1 Surgical procedure

The principles of surgical treatment of refractory or severe pressure injuries are well described for the adult population^16^ and were followed also in our paediatric patients.

They include thorough debridement of all nonviable tissue, removal of all foreign material, copious irrigation, suction drainage positioning, flap coverage, and adequate post‐operative measures and monitoring. All the nonviable or doubtable viable tissue was removed en‐block. Bony prominences responsible for the pressure injuries were removed if nonviable or infected; otherwise, they were reduced in volume and modelled in shape with scalpels or burrs.

If the cause of the pressure lesion was a medical device or other foreign material, we considered it contaminated, because exposed and, if possible,^17^ we removed it. After debridement, the defect was re‐assessed to evaluate and choose the available reconstructive options.

Negative pressure wound‐therapy was sometimes used, to prepare the wound bed, reducing bacterial contamination, removing secretions, promoting granulation, and reducing the size of the defect.^18^

### 2.2 Several flaps were used for reconstruction

In most cases, we used a pedicled flap, and only rarely microsurgical free flaps.

A pedicled flap is a portion of tissue that maintains its vascular supply by preservation of the vessels entering and exiting the flap and that are called pedicle of the flap. When a pedicled flap is harvested from the area surrounding the defect to be covered, it is called local flap, while if it is harvested at some distance from the defect that it is reached by means of a longer pedicle, it is called regional flap. In our study, in most cases local flaps were sufficient to obtain a safe and durable coverage, regional flaps being seldom necessary. A microsurgical flap, very rarely used in our study, cannot reach the defect by means of a pedicle because it is harvested too far from it. Its vessels are therefore divided, the flap is transferred into the defect and the artery and vein are anastomosed with microsurgical technique to an artery and vein close to the defect.

A suction drain was always positioned on the surgical site and left in place for several days and up to 2 weeks.

## 3 RESULTS

We treated 18 children and adolescents aged 1–17 years with pressure injuries Stages 3 and 4, who required 20 flaps and three re‐interventions to treat recurrences or new injuries. Follow‐up ranged from 19 months to 13 years.

Details on the patients are reported in Table 1.

**TABLE 1:** Details of our patients characteristics, treatment, follow up and recurrences

Case	Age (years), sex (M–F)	Site	Comorbidities	Paraplegia	Medical device exposure	Stage	Time from onset	Culture	Medical therapy	Surgical therapy	FU months	FU Recurrence (and site)	FU Recurrence treatment	FU Other complications	Other events
1	13, M	Thoracic spine, exposed bone fixation devices	‐ Severe developmental delay ‐ Spinal instrumentation 2 years before for severe kyphosis		YES	4	Six months	Multibacterial	Intravenous antibiotics based on antibiogram	Debridement and cover with inferior trapezius muscle flap and lateral relaxing skin incision (Impossible to remove spinal instrumentation because of danger of collapse)	Four years and 6 months	NO			
2	14, F	Medial plantar	‐ Congenital myelomeningocele			3	Four months	Contaminants	‐	Debridement and cover with dorsalis pedis flap	Eight years and 10 months	YES Two years later Ipsilateral heel with osteomyelitis	Radical debridement and direct closure		
3	8, M	Temporal, exposed cochlear implant	‐ Congenital neurosensorial deafness		YES	4		*Staphylococcus aureus*	Intravenous antibiotics based on antibiogram	Implant salvage with TPF cover	Eight years and 8 months	NO			
4	15 months, F	Hypogastrium, exposed pacemaker	‐ Congenital cardiac anomaly (dilated cardiomyopathy with complete AV block)		YES	4		Multibacterial	Intravenous antibiotics based on antibiogram	Replacement of pacemaker and cover with rectus muscle advancement and skin rotation flap	Nine years and 10 months	NO			
5	13, M	Sacro‐gluteal	‐ Paraplegia ‐ Congenital myelomeningocele	YES		4	Two years	Multibacterial		SGAP perforator flap (propeller)	Six years, 3 months	NO			
6	16, M	Sacro‐gluteal	‐ Congenital myelomeningocele	YES		4	Eighteen months	Multibacterial		Rotation flap (SGAP)	Seven years and 5 months	NO			
7	12, F	Sacro‐gluteal, left	‐ Post‐surgical			4	Three days			Rotation flap	Three years	None	None	–	
8	8, F	Sacrococcygeal	‐ Neuroblastoma ‐ Paraplegia	YES		4		*MRSA*, *Escherichia coli, Pseudomonas aeruginosa, Candida albicans*		Coccygectomy and rotation flap	Seven years	NO		None	
9	16, F	Lateral foot	‐ Congenital myelomeningocele	YES		4	> Three years	*Proteus mirabilis, Enterobacter* spp.	None	Radical debridement and free ALT	159	NO	None	None	None
10	15, M	Ischial	Paraplegia (post‐traumatic)	YES		4	Two years	*Pseudomonas aeruginosa*	None	Radical debridement, partial ischiectomy, pedicled TMG	154	YES Trochanteric, Bilateral after 9 and 12 years	Radical debridement and Pedicled Vastus Lateralis flap	Postoperative infection treated with culture guided Intravenous antibiotics	None
11	14, F	Sacral	Paraplegia (Post‐traumatic)	YES		4	Eighteen months	Multibacterial	None	Radical debridement, partial sacrectomy, pedicled SGAP	53	NO	None	None	Temporary venous congestion. Flap delayed and transferred after 3 days. Uneventful afterwards.
12	17, M	Ischial (bilateral)	Tetraplegia (Post‐traumatic)	YES	NO	4	Six months	Multibacterial	None	Radical debridement, posterior thigh flap (right side) and pedicle IGAP (left side) in two stages	Nine years	YES ischial right side after 8 years	Conservative treatment until now (pressure relief and dressings)	None	None
13	4, F	Lumbosacral	Spina bifida, chronic wound after laminectomy L5, transection filum terminale tethered cord	NO	NO	4	Two months		None	Radical debridement, 2 local fasciocutaneous rotation flaps	Four years	YES osteomyelitis S2 with fracture and overlying cellulitis	Intravenous ceftriaxone and oral flucloxacilline	None	None
14	13, F	Thoracic, exposed bone fixation devices	Spina bifida, surgery for severe kyphoscoliosis	YES	YES	4	Four months	*Pseudomonas aeruginosa*	Ciprofloxacin	Radical debridement, pedicled perforator flap (free style)	Eight years	YES, 1 year post flap coverage	removal of medical device + fistulectomy and closure	None	None
15	12,F	Pretibial, exposed bone fixation devices	Congenital myelomeningocele, surgery for tibial rotation correction	NO	YES	4	Two months	Negative	None	Radical debridement, posterior tibial perforator flap + graft + negative pressure therapy	Twenty‐four months	None			Flap liposuction for aesthetic improvement 2 years postoperatively
16	15,M	Ischial (right)	Congenital myelomeningocele	YES	NO	4	Eleven months	*Streptococcus pyogenes*	IV antibiotics adapted to antibiogram	Radical debridement including bone, posterior thigh muscle flap.	Nineteen months	None		Hematoma and infection postoperatively Drainage, IV antibiotics and conservative treatment.	
17	1,F	Temporal, exposed cochlear implant	Congenital neurosensorial deafness	NO	YES	4		*Staphylococcus aureus*	IV antibiotics adapted to antibiogram	Implant salvaged with TPF flap cover	Five years	None			
18	2,M	Temporal, exposed cochlear implant	Congenital neurosensorial deafness	NO	YES	4		Negative	None	Implant salvage with TPF falp cover + split thickness skin graft	Five years	None			

Abbreviations: ALT, anterolateral thigh; AV, atrio‐ventricular; FU, follow‐up; IGAP, inferior gluteal artery perforator; SGAP, superior gluteal artery perforator; TPF, temporo‐parietal fascia flap; TMG, transverse musculocutaneous gracilis.

Eight patients were wheelchair‐dependent, and 12 had sensory impairment at the ulcer site. One patient developed a PI during the hospital stay, following abdominal surgery under general anaesthesia for peritonitis.

Osteomyelitis that required prolonged antibiotic therapy was present under one injury (Case 8, coccyx) and in two other cases (Cases 2 and 13) complicated a new injury during the follow‐up period.

Complications were few and included one postoperative infection treated with culture‐guided intravenous antibiotics in one case (Case 10), one postoperative infected hematoma (Case 16) that required drainage, intravenous antibiotics and conservative treatment.

### 3.1 Clinical cases

We describe five cases illustrative of treatment in different body areas, listing reconstructive options alternative to our choice.

#### 3.1.1 PI on the thoracic spine

*Case 1*: The patient was a 13‐year‐old male with severe developmental delay due to an unclassified syndrome. Two years before he had undergone orthopaedic correction of severe kyphosis with titanium bars. At arrival at our centre, he presented with a Stage 4 PI, localized over the thoracic spine that had developed 6 months previously. Wound culture showed multibacterial growth that was treated with intravenous antibiotics according to the antibiogram.

**FIGURE 1:**
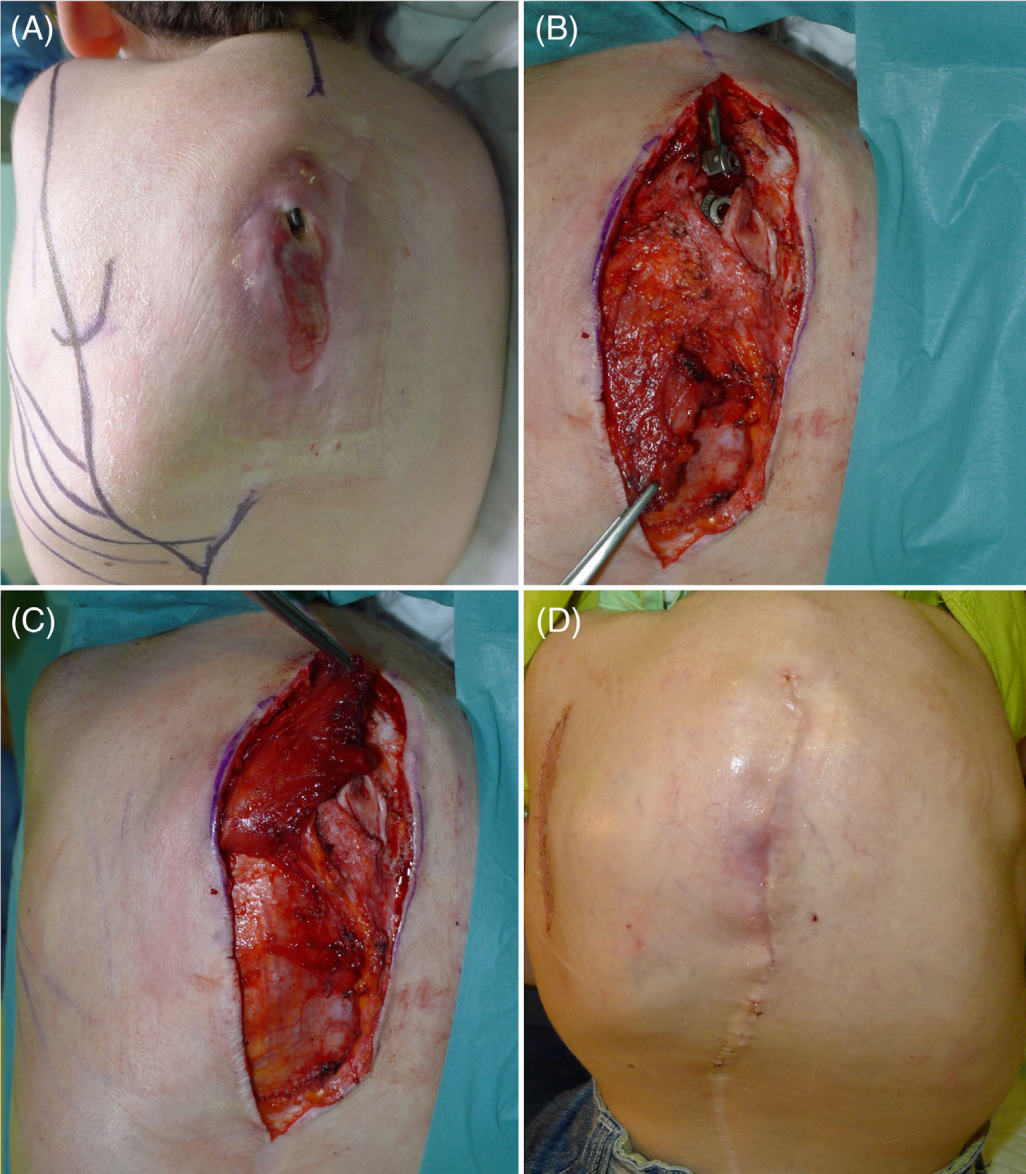
(A) Pressure injury, Stage 4, localized over the thoracic spine with exposed titanium bar. (B) Radical debridement of the pressure injury, isolation of inferior trapezius. (C) Coverage of titanium bar with inferior trapezius turn‐over flap. (D) Skin closure obtained with a bipedicled fascio‐cutaneous flap through a lateral relaxing incision that was skin grafted [Color figure can be viewed at wileyonlinelibrary.com]

The spinal instrumentation could not be removed because of the danger of vertebral collapse. Surgery included debridement, multiple sampling for microbiology, and coverage of the exposed hardware with an inferior trapezius turnover flap. Skin closure was obtained with a bipedicled fascio‐cutaneous flap through a lateral relaxing incision that was skin grafted. This prevented the formation of a skin scar overlying the maximal pressure point (Figure 1).

An alternative option would have been a latissimus dorsi myocutaneous pedicled flap, which would have had the advantage of good muscular coverage but the disadvantage of more invasive surgery and donor site morbidity. A second alternative would have been a local fascio‐cutaneous flap, carrying the advantage of less donor site morbidity with muscle preservation but with the disadvantage of a thinner flap, at a higher risk of a recurrence.

#### 3.1.2 Temporal device associated PI

**FIGURE 2:**
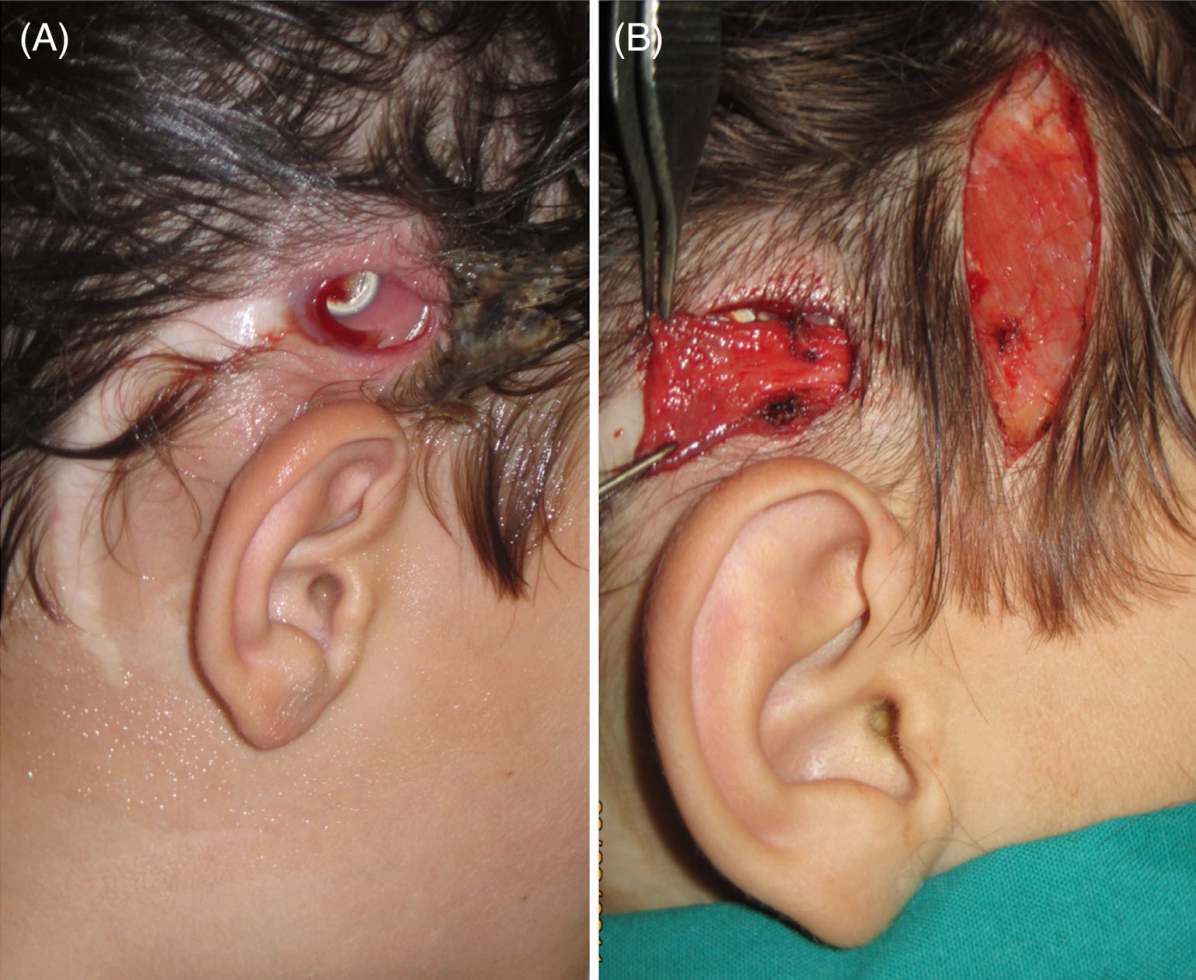
(A) Pressure injury with exposure of a cochlear implant. (B) Wound debridement and coverage with temporalis fascia flap. The scalp was mobilized and the wounds closed primarily [Color figure can be viewed at wileyonlinelibrary.com]

*Case 3*: The patient was an 8‐year‐old male with congenital neurosensory deafness who came to our attention with a PI over the exposed cochlear implant (Figure 2).

Wound culture grew *Staphylococcus aureus*. Medical treatment included intravenous antibiotics based on the antibiogram. Surgical treatment included wound debridement and copious irrigation of the tissues with saline solution. To increase the chance of salvaging the implant, we covered it with the temporo‐parietal fascia flap, a local axial flap with excellent perfusion. The scalp was then mobilized to cover the flap, closing the wound by first intention.

An alternative would have been a scalp rotation flap, with less donor site morbidity but less protection of the savaged implant.

#### 3.1.3 Sacro‐coccygeal

**FIGURE 3:**
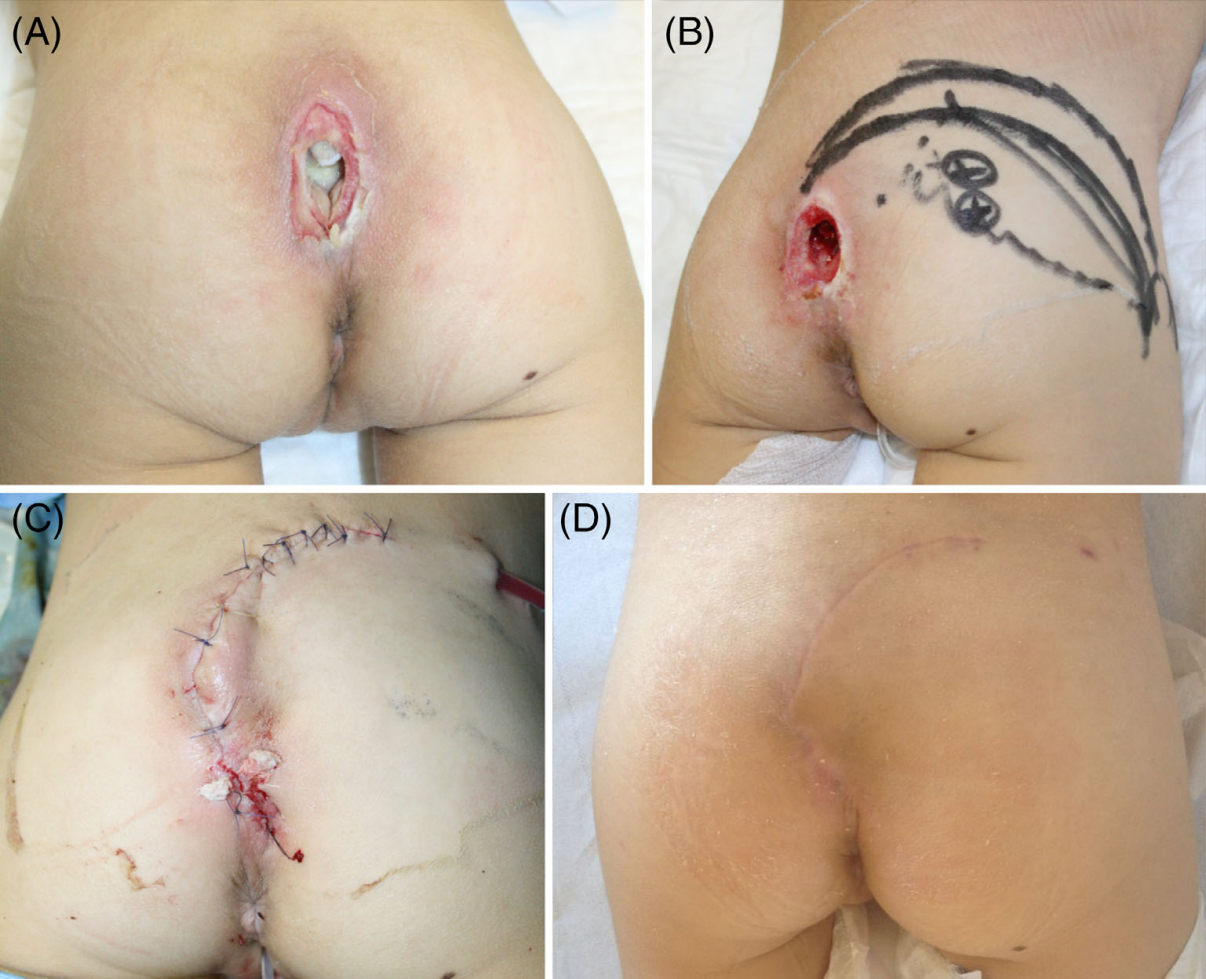
(A) Purulent sacrococcygeal pressure injury Stage 4 in an 8‐year‐old girl with paraplegia secondary to neuroblastoma. (B) Debridement of the pressure injury with coccygectomy due to multibacterial osteomyelitis. On the right gluteus, we marked two variations of a rotation flap. Two perforators were audible at Doppler. (C) Immediate postoperative result after adipo‐cutaneous rotation flap. The tip of the flap was de‐epithelized and buried in the cavity to fill the tissue defect and to increase the strength of the repair. (D) Four months after surgery: a good quality scar and no sign of recurrence [Color figure can be viewed at wileyonlinelibrary.com]

*Case 8*: An 8‐year‐old girl that had developed paraplegia due to neuroblastoma, presented with a sacrococcygeal purulent ulcer. (Figure 3).

Microbiological cultures grew methicillin sensitive *S. aureus* from the wound and *Escherichia coli*, *Pseudomonas aeruginosa* and *Candida albicans* from the coccygeal bone. A diagnosis of multibacterial osteomyelitis was therefore made. Debridement required coccygectomy that left an 8 cm × 4 cm × 4 cm soft tissue defects.

We chose for reconstruction a classical adipo‐cutaneous rotation flap. The tip of the flap was de‐epithelized and buried in the cavity to fill the tissue defect and to increase the strength of the repair. Among the alternatives described in the literature for this kind of PI is V to Y advancement flaps, keystone flaps and superior gluteal artery perforator (SGAP) flap. A rotation flap, however, leaves smaller scars located in a better position, and may be used again, in case of recurrence.

#### 3.1.4 Plantar injury

**FIGURE 4:**
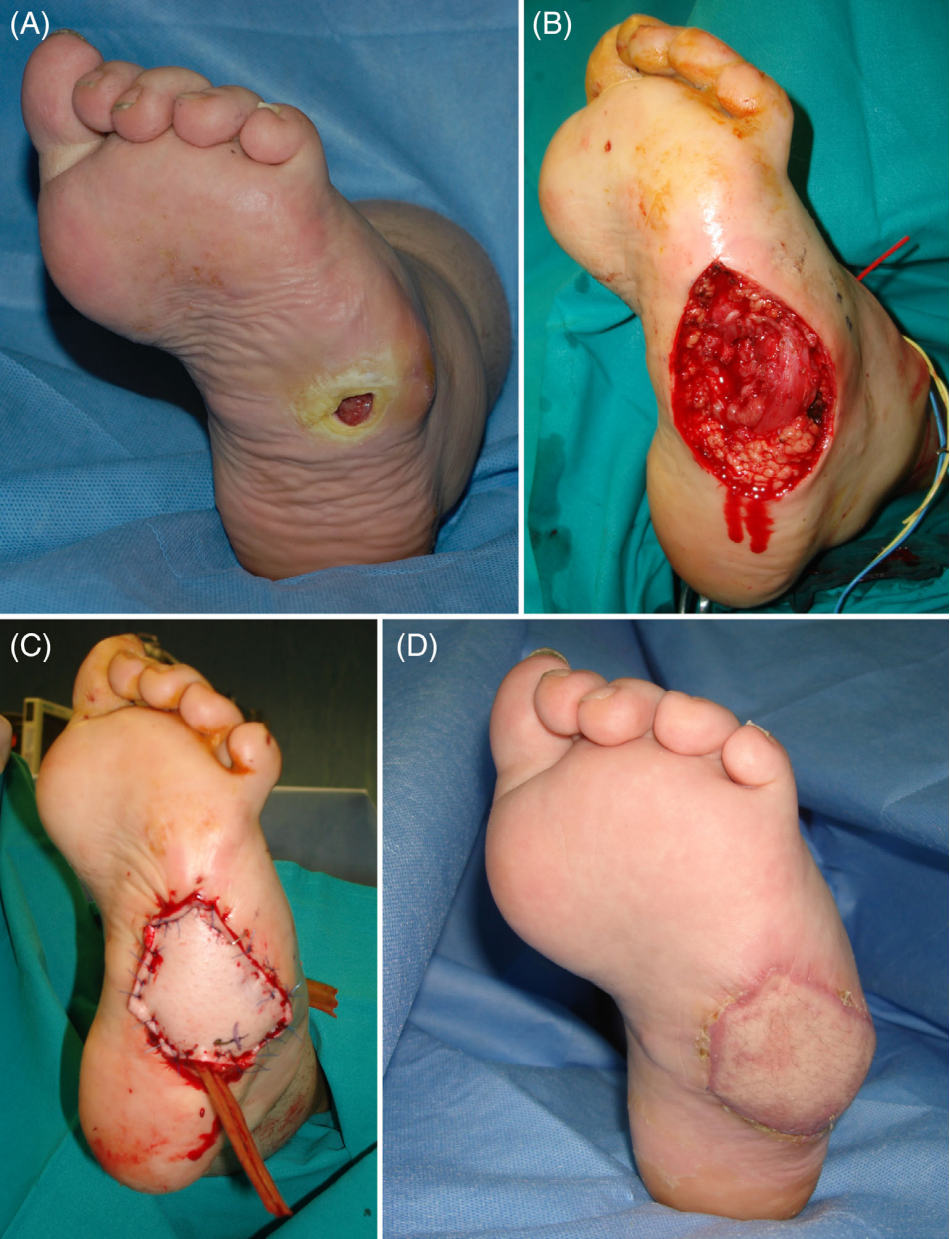
(A) Lateral plantar pressure in a 16‐year‐old girl with compromised ambulation due to congenital myelomeningocele treated in the early postnatal period. (B) Extensive debridement of the pressure injury reaching healthy tissue. (C) Reconstruction of the lateral plantar aspect of the foot with ALT free flap. (D) Result at 2 months after surgery [Color figure can be viewed at wileyonlinelibrary.com]

*Case 9*: A 16‐year‐old girl, treated in the early postnatal period for congenital myelomeningocele developed a lateral plantar PI due to her compromised ambulation. (Figure 4) In this case, to provide healthy tissue from a zone outside the foot, limiting the scars in this extremity, we chose microsurgical reconstruction. The antero‐lateral thigh flap has an excellent donor site and does not cause functional sacrifice.

A potential alternative would have been an instep pedicled flap. A less invasive treatment with negative pressure, followed by skin graft would not have been indicated in a site exposed to pressure because of the risk of future ulceration.

#### 3.1.5 Bilateral ischial injury

**FIGURE 5:**
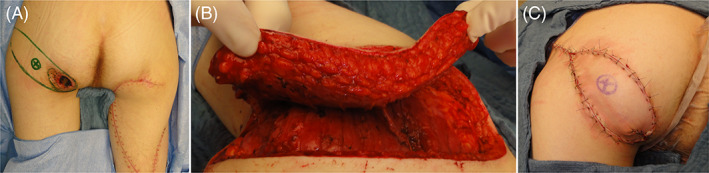
(A) Bilateral ischiatic pressure injuries in a 17‐year‐old boy with tetraplegia due to a cervical spine injury after a diving accident. The injuries were treated separately with an interval of 6 weeks between the two surgical procedures. The right one was treated with a posterior thigh advancement flap (‘hatchet’). (B) The pedicled inferior gluteal artery perforator flap was harvested to treat the left ischiatic injury. (C) Immediate postoperative result of the left pressure injury reconstruction (IGAP V‐Y advancement flap) [Color figure can be viewed at wileyonlinelibrary.com]

Case 12 was a 17‐year‐old boy with tetraplegia due to a cervical spine injury after a diving accident. C4–C5 fusion was performed, but due to the prolonged immobilization in the intensive care unit, he developed bilateral Stage 4 ischial pressure sores. Radical debridement and closure with a posterior thigh advancement flap on the right side and a pedicled inferior gluteal artery perforator flap (IGAP) on the left side were performed in two stages with 6 weeks in between the two surgical procedures. (Figure 5).

Wound healing was uneventful and he was discharged after an intensive rehabilitation program. Regular follow up as an outpatient showed no recurrence until 9 years postoperatively, when a Stage 2 PI recurrence, that was treated conservatively, developed on the right side.

### 3.2 Recurrences

During follow‐up lasting 19 months to 13 years, two of our patients (Cases 12 and 13) developed a recurrence, while Cases 2 and 10 developed new injuries in a different anatomical site. (Table 1).

Case 2 presented with a new injury in the heel after having been operated the first time in the instep. Although this cannot be considered as a true recurrence, the change in pressure point after the first flap probably influenced the site of the new injury.

## 4 DISCUSSION

Paediatric patients, especially neonates and infants, are vulnerable to PI formation that, although relatively infrequent, deserve adequate treatment.

The evidence‐based recommendations for the prevention and treatment of pressure ulcers, published under the auspices the National Pressure Injury Advisory Panel (NPIAP) in collaboration with the European Pressure Ulcer Advisory Panel (EPUAP), are now shared also by the Pan Pacific Pressure Injury Alliance (PPPIA), to develop a uniform behaviour by health professionals worldwide. (European Pressure Ulcer Advisory Panel and National Pressure Ulcer Advisory Panel, 2014). An updated NPIAP White paper on neonates and infants was published in 2019.^12^

Early diagnosis, accurate care, changing position, maintaining scrupulous hygiene, and the use of specific devices to dissipate pressure or change pressure points are the first and easiest ways to intervene. Compliance is often difficult to obtain in children and adolescents, but the problem can be overcome involving their parents, who are almost always of great support.

Baharestani & Ratliff, and, more recently, Delmore et al. reviewed data on the treatment of pediatric PIs and the specific factors that make neonates and children vulnerable.^12^, ^13^

Superficial pressure injuries (Stages 1 and 2) can heal spontaneously, but, in our experience, deep pressure injuries (Stages 3 and 4) and unstageable PI that do not heal spontaneously, benefit from surgical intervention.

Our series probably included selected patients who did not respond to non‐invasive treatment.

The choice between conservative treatment and surgery for a PI depends on a complete evaluation of the ulcer, as well as of the patient's physical and mental state.

When surgery is needed, adequate patient preparation (control of comorbidities), thorough debridement of the ulcer, choice of the most suitable reconstruction technique, patient's compliance throughout the entire treatment period, professional postoperative support, and sufficient pressure relief are imperative for success.

Antibiotic therapy, which is mandatory in the presence of systemic spread of the infection, or of osteomyelitis, is only occasionally necessary in treating PIs.^19^, ^20^

After debridement, soft tissue reconstruction is safe, even over a contaminated wound.^21^ Several surgical techniques have been proposed, including direct closure and skin grafting (rarely indicated) and, more importantly, local, regional, and microsurgical free flaps.^12^, ^13^, ^18^, ^22^, ^23^

Although reconstructive techniques used in the paediatric population do not differ significantly from the ones used in the adult, the different characteristics of soft tissues in children, must be considered.

In children, the subcutaneous tissues are firmer and therefore more difficult to mobilize than in the elderly, while skin and dermis are more elastic.^24^ In children, the greater softness and elasticity of the skin and dermis are of help when planning and harvesting a flap for reconstruction, allowing smaller incisions and possibly reducing wound tension. In addition, the vessels are generally healthy, and skin perfusion richer, facilitating the success of regional or free flaps.^25^

In treating PI, it is important to use a single flap, planned in such a way as not to interfere with the need for future flaps, especially in children affected by chronic conditions, who face a life–long risk of developing PIs. As a consequence, among the different reconstructive techniques used in our paediatric population, rotation flaps were the first choice for sacral/gluteal pressure sores. Rotation flaps require, especially in children, limited undermining, leaving the gluteal area available for future flaps. They cause minimal donor site morbidity and leave the scar in a position that can be usually covered by clothes.^26^

The rotation flaps (Cases 6–8 and 13) were harvested with a traditional technique (Cases 7, 8 and 13) or preserving the encountered perforators of adequate size (Case 6). These need to be isolated and freed for a few centimetres into the muscle to allow the movement of the flap. As reported in the literature, local perforator flaps have been extensively used to reconstruct pressure injuries, with different donor sites and skin island shapes.^27^

According to Lin and Yang,^23^, ^27^ the flap becomes ‘a reusable perforator‐preserving gluteal artery‐based rotation flap’ with the already cited advantages of the traditional rotation flap and the safety of a well‐perfused perforator flap.

The possibility to reuse these flaps in case of PI recurrence has been described.^23^

Therefore, due to their advantages,^28^ we used pedicled perforator flaps, in different anatomical sites, in Cases 5, 11,12, 14 and 15.

As discussed by Rethlefsen,^29^ foot pressure injuries develop in approximately 10% of paediatric patients suffering from spina bifida. In our study, we treated two such patients with different flaps. In one case with a dorsalis pedis (Case 2) and in one (Case 9) with a free antero lateral thigh (ALT) flap.

The microsurgical ALT flap was chosen because it is considered the best option for lower limb reconstruction in children^30^ being reliable, having a relatively thick dermis and the advantage that the donor site can be hidden. Local flaps from weight‐bearing surfaces of the same foot were not considered necessary in this case because the patient was not ambulatory, and were avoided to preserve the area in case of new or recurrent PIs.

According to the literature, medical devices are frequently (38.5%–90%) involved in the aetiology of PIs (now specifically called medical device‐related PIs) and affected patients are usually younger. In their presence, prevention strategies seem to be particularly important.^15^

Seven cases of PIs with exposed medical devices were included in our study. The devices were osteosynthesis material in Cases 1, 14 and 15, a cochlear implant in Cases 3, 17 and 18, a pacemaker in Case 4.

Different flaps have been used for each of them, always aiming at providing secure perfusion and durable padding with, in most cases, a double layer of closure. Trapezius muscle flap in Case 1, fascia temporalis in Case 3, 17 and 18, rectus abdominis muscle in Case 4, all but one (Case 18) covered by a separate overlying adipo‐cutaneous layer. The devices have been successfully maintained in site in all cases except for Patient 4, in whom a change of device and location became necessary.

Although the recurrence rate of PIs is known to be high in adults, the few data reported in children are not uniform. Singh et al.^15^ reported an overall PI recurrence rate of 5% (one of 20 PIs) while in Firriolo's series^14^ there was a 42% recurrence rate in ulceration after flap reconstruction, that correlated with a preoperative history of noncompliance with conservative therapy. (Table 2).

**TABLE 2:** Comparison between our study and two other published reports in the literature on surgical treatment of pediatric pressure sores

	Singh et al., 2002	Firriolo et al., 2018	Pignatti et al., 2020
Study type	Retrospective	Retrospective	Retrospective
Indication for surgery	Grades III and IV pressure injuries	Stages III and IV pressure ulcers	Stages 3 and 4 pressure injuries
No. of patient	19	24, 7 Female and 17 male	18, 10 Female and 8 male
No. of pressure injuries	25	30	20
Patients with follow‐up	15 (79%), 7 female and 8 male	14	18
Pressure injuries with follow‐up	20 (80%)	N.A.	20 (100%)
Mean age (range)	16,2 (9–25) years	14,6 (3,7‐20,6) years	10,8 (1–17) years
Mean postoperative follow‐up (range)	5,3 years (11 months ‐ 11 years)	N.A.	6,7 years (19 months–13 years)
Pressure injuries risk factors	‐ Spina bifida (12 patients) 80% ‐ Spinal cord injury (two patients) 13% ‐ Cord tumour (one patient) 7%	‐ Myelomeningocele (16 patients) 67% ‐ Paraplegy secondary to various aetiologies (four patients) 17% ‐ Spastic quadriplegic cerebral palsy (two patients) 8% ‐ Lipomeningocele (Two patients) 8%	‐ Para/tetraplegia (8 patients) 44% ‐ Myelomeningocele (six patients) 33% ‐ Neurosensorial deafness (three patients) 17% ‐ Spina bifida (two patients) 11% ‐ Sever development delay (one patient) 6% ‐ Spinal instrumentation (one patient) 6% ‐ Congenital cardias anomaly (one patient) 6% ‐ Previous major surgery ‐ Neuroblastoma (ine patient) 6%
Wheelchair dependency	N.A.	23. ‐ Only one patient was ambulatory	8. ‐ Twelve patients had sensory impairment at the ulcer site.
Average wound duration	11.3 months	N.A.	N.A.
Pressure injuries distribution	‐ Seven sacral ‐ Nine ischial ‐ Three trochanteric ‐ One Iliac crest	‐ Fifteen ischial ‐ Eight Sacral ‐ Three feet ‐ Two coccyx ‐ Two trochanteric ‐ Two gibbus deformities ‐ One involved both ischium and sacrum ‐ One affected both sacrum and coccyx	‐ Four ischial ‐ Three temporal ‐ Six sacral ‐ Two thoracic ‐ Hypogastrium ‐ Pretibial ‐ Medial plantar ‐ Lateral foot
Reconstructive surgery technique (including recurrences)	‐ Twenty‐three myocutaneous flaps	‐ Thirty‐seven muscle or musculocutaneous flaps ‐ 15 Fasciocutaneous flap	‐ Eighteen fasciocutaneous flaps ‐ Five myocutaneous flap
The mean hospital stay (range)	9.1 (7–14) days	N.A.	N.A.
Site‐specific recurrence rate	5%	42%	11%
Previous patient who developed a new sore	20%		11%

Of our 18 patients followed for up to 13 years, two developed a recurrence (Cases 12 and 13) and two patients (Cases 2 and 10) developed new injuries in a different anatomical site and needed to be re‐treated.

Contrary to Firriolo's study,^14^ where wheelchairs and other equipment for many of the patients were inadequate and in bad condition, and medical care was inconsistent because of self‐reported insurance coverage limitations, in our countries, the public health systems supply equipment and free medical follow‐up.

## 5 CONCLUSIONS

Flap reconstruction is usually beneficial for the treatment of PIs in the paediatric patients in whom conservative therapy was not sufficient. However, when surgical repair is necessary. We found in our multicentre retrospective study that long‐term success could be achieved with accurate debridement, tailored reconstructive surgery and prevention of new injuries. In our experience, the prevalence of recurrence was lower than previously reported.

## CONFLICT OF INTEREST

None.

## Data Availability

Data available on request due to privacy/ethical restrictions
